# Unlocking Low‐Melting‐Point and High‐Conductivity Molten Salts via High‐Entropy Strategy with Demonstration in Thermal Batteries

**DOI:** 10.1002/advs.76847

**Published:** 2026-09-03

**Authors:** Binchao Shi, Ting Sun, Fushan Yu, Yi Zhang, Gaowu Wang, Ting Quan, Yan‐li Zhu

**Affiliations:** ^1^ State Key Laboratory of Explosion Science and Safety Protection Beijing Institute of Technology Beijing China; ^2^ Xi'an North Qinghua Electromechanical Co. Ltd Xi'an China; ^3^ BYD Lithium Battery Co., Ltd. Shenzhen China

**Keywords:** high‐entropy, low melting point, molten‐salt electrolyte, thermal battery, ultrafast activation

## Abstract

The development of advanced molten salt electrolytes for thermal batteries has long been hindered by an intrinsic trade‐off between low melting point and high ionic conductivity, posing a fundamental limitation to their performance optimization. To address this challenge, this work reports a high‐entropy design strategy that breaks this long‐standing constraint by leveraging configurational complexity to simultaneously modulate thermodynamic and transport properties. By constructing a quinary LiF‐LiCl‐LiBr‐KBr‐CsBr molten salt system, the introduction of multiple ionic species generates substantial lattice distortion and high configurational entropy, leading to a markedly reduced melting point of 229.5°C and 1.19 S cm^−1^ at 350°C. When implemented in thermal batteries, a prototype battery achieves ultrafast activation within 64 ms. This work establishes high‐entropy design as a general pathway to advanced electrolytes for high‐temperature electrochemical applications.

## Graphical abstract

A high‐entropy strategy is developed to break the trade‐off between low melting point and high ionic conductivity in molten salt electrolytes. By incorporating CsBr and LiF into the LiCl‐LiBr‐KBr system, the quinary LiF‐LiCl‐LiBr‐KBr‐CsBr electrolyte achieves a record‐low melting point of 229.5°C while maintaining a high conductivity of 1.19 S cm^−1^ at 350°C.

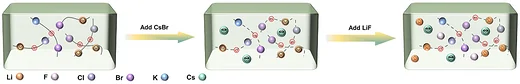

## 1 Introduction

The development of high‐performance high‐temperature electrochemical devices is fundamentally governed by the availability of advanced electrolyte materials [1, 2, 3, 4, 5, 6]. Among them, molten salts serve as the cornerstone for a wide range of applications in thermal batteries, liquid metal batteries, and high‐temperature fuel cells due to their high ionic conductivity, wide electrochemical window, and excellent thermal stability [7, 8, 9, 10, 11, 12]. Nevertheless, the persistent challenge in the field of molten salt chemistry is the intrinsic trade‐off between low melting point and high ionic conductivity. Lowering the melting point is essential for reducing the activation energy and thermal load, but conventional design strategies, such as the introduction of single species such as large‐radius cations (Cs^+^) or soft anions (I^−^), typically result in the disruption of ion transport pathways, leading to decreased ionic conductivity and compromised physicochemical stability [13, 14, 15, 16, 17]. This inherent coupling between thermodynamic properties and ion transport behavior presents a persistent challenge for the rational design of advanced molten salt electrolytes.

In recent years, high‐entropy materials have shown disruptive potential in multiple fields of materials science, due to their unique synergy and entropy‐driven mechanisms [18, 19, 20, 21, 22, 23, 24]. By introducing multiple principal components, these systems leverage configurational entropy to stabilize disordered structures, suppress phase separation, and modulate local chemical environments [25, 26, 27, 28, 29]. Beyond thermodynamic stabilization, the high‐entropy effect can profoundly influence ion transport by altering coordination structures and dynamic interactions among species. This strategy has recently demonstrated significant advantages in the field of lithium‐ion batteries and has been successfully applied to electrolyte design, effectively enhancing structural stability and ion transport kinetics [30, 31, 32, 33, 34]. For instance, Duan et al. developed a high‐entropy polymer electrolyte incorporating TFSI^−^, FSI^−^, DFOB^−^, and BF_4_^−^ anions to construct a high‐entropy Li^+^ coordination environment. The synergistic effect of the multiple anions significantly reduced the binding energy between lithium ions and the polymer chains, facilitating Li^+^ migration. Additionally, a steric hindrance effect helped form dynamic ion‐conducting channels. As a result, the electrolyte achieved a high ionic conductivity of 0.238 mS cm^−1^ and a lithium‐ion transference number of 0.707. Batteries assembled with this electrolyte demonstrated excellent long‐term cycling stability [35]. Similarly, a five‐component high‐entropy electrolyte (containing LiFSI, LiTFSI, LiPF_6_, LiDFOB, and LiNO_3_) was designed by Li et al. By leveraging the entropy effect to suppress lithium‐ion cluster aggregation and reduce polymer crystallinity, the ionic conductivity of the electrolyte at ‐20°C was increased by 8.5 times. Furthermore, it promoted the formation of a stable high‐entropy SEI film, significantly enhancing the low‐temperature cycling performance of lithium metal batteries [36]. The ability of high‐entropy materials to synergistically optimize multiple properties through compositional design provides an inspiring new approach to addressing the trade‐off challenges between melting point and conductivity in molten salt electrolytes.

Guided by this rationale, we extend high‐entropy compositional engineering to molten salt electrolytes and propose a multi‐component design to decouple melting behavior from ionic transport. By precisely regulating molecular diversity, a quinary LiF‐LiCl‐LiBr‐KBr‐CsBr molten salt is developed based on the conventional low‐melting LiCl‐LiBr‐KBr system. The incorporation of multiple ionic species introduces significant lattice distortion and increases configurational entropy, thereby destabilizing ordered arrangements and lowering the melting point. Meanwhile, the synergistic combination of CsBr and low‐molecular‐weight LiF plays a dual role, in which CsBr weakens interionic Coulombic interactions, while LiF enhances structural disorder and increases the Li^+^ molar fraction, collectively facilitating ion migration. Through this compositional design, the conventional coupling between thermodynamic and transport properties is effectively mitigated. As a result, the LiF‐LiCl‐LiBr‐KBr‐CsBr system achieves an ultra‐low melting point of 229.5°C (90°C lower than the commercially used LiCl‐LiBr‐KBr molten salt) and a stable ionic conductivity of 1.19 S cm^−1^ at 350°C (two orders of magnitude higher than that of nitrate‐based systems at comparable temperatures). Furthermore, the molten salt exhibits low fusion enthalpy (18.7 kJ mol^−1^) and specific heat capacity, which significantly decrease the thermal energy required for phase transition and contribute to improved energy efficiency during operation.

To further evaluate its practical applicability, the high‐entropy molten salt is employed in thermal batteries, which impose stringent requirements on electrolyte performance, including rapid activation and stable discharge under high‐temperature conditions [37, 38, 39]. Benefiting from the rapid establishment of effective ionic conduction pathways, the prototype thermal battery demonstrates fast activation within 64 ms and stable discharge for 82 s at a current density of 25 mA cm^−2^. Overall, this work underscores that high‐entropy compositional engineering is an effective and versatile strategy for tailoring molten salt electrolytes, enabling the simultaneous optimization of melting behavior and ionic transport. This approach provides new insights into the design of advanced electrolyte materials and opens up opportunities for next‐generation high‐temperature electrochemical systems.

## 2 Results

### 2.1 The Design Strategy of Molten Salts

**FIGURE 1:**
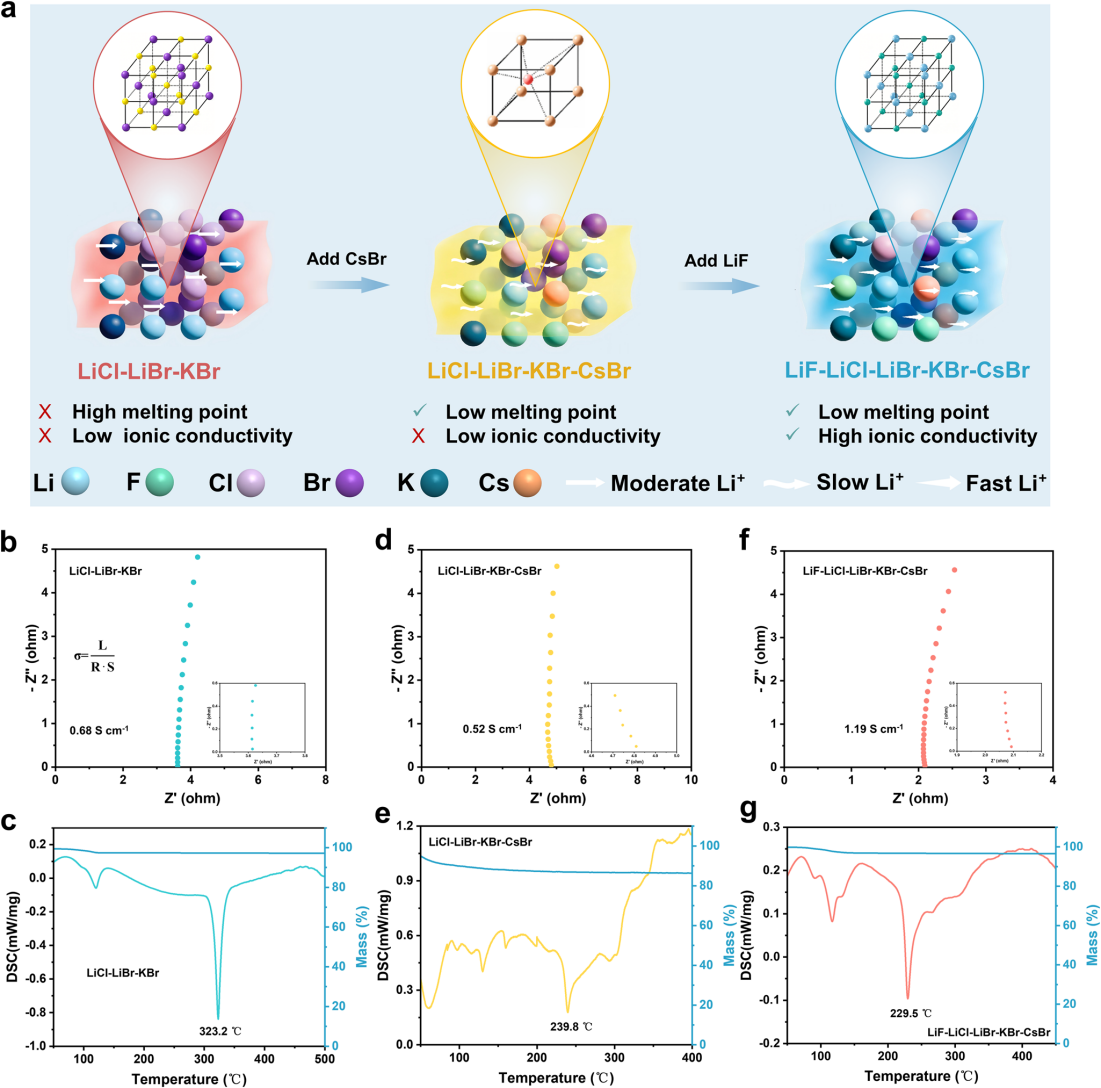
Design strategy and characterization of molten salt electrolytes. (a) Schematic illustration of the design strategy for developing low‐melting‐point, high‐ionic‐conductivity molten salt based on LiCl‐LiBr‐KBr. (b, c) Nyquist plots and TG‐DSC of LiCl‐LiBr‐KBr (25‐37‐38 mol%). (d,e) Nyquist plots and TG‐DSC of LiCl‐LiBr‐KBr‐CsBr (25‐37‐25‐13 mol%). (f,g) Nyquist plots and TG‐DSC of LiF‐LiCl‐LiBr‐KBr‐CsBr (13‐25‐37‐12‐13 mol%).

The molten salt design is based on the widely used LiCl‐LiBr‐KBr ternary system. In this foundational system, LiCl and LiBr serve as the primary lithium sources, establishing the essential pathways for lithium‐ion conduction [40]. The design rationale of the developed molten salt is presented in Figure 1a. To simultaneously reduce the melting point and enhance the electrical conductivity, a stepwise high‐entropy design strategy was employed. A series of molten salts with systematically varied compositions were prepared (Table S1), and their thermal behaviors were evaluated using DSC. As shown in Figures S1 and S2, at a specific ratio, a pronounced synergistic effect between LiF and CsBr emerges near specific compositions, resulting in a significantly depressed eutectic temperature and a narrowed melting range. The complete component screening results and corresponding melting points are summarized in the Supporting Information.

The baseline LiCl‐LiBr‐KBr system exhibits a melting point of 323.2°C and an ionic conductivity of 0.68 S cm^−1^ at 350°C (Figure 1b,c). Substituting KBr with CsBr introduces stronger lattice distortion due to the larger ionic radius of Cs^+^, reducing the melting point to 239.8°C but at the expense of ionic conductivity (Figure 1d,e), highlighting the intrinsic trade‐off. To overcome this trade‐off, LiF was strategically incorporated to construct the quinary LiF‐LiCl‐LiBr‐KBr‐CsBr system, in which LiF increases the lithium‐ion concentration while enhancing structural disorder. Coupled with the configurational entropy arising from multi‐component mixing, the system achieves simultaneous optimization of thermodynamic and transport properties. The optimized composition exhibits a melting point of 229.5°C and a high ionic conductivity of 1.19 S cm^−1^ at 350°C (Figure 1f,g). This breaking of the trade‐off between the melting point and conductivity, combined with the excellent thermal stability confirmed by TG, validates the superiority of the entropy‐engineered approach in developing high‐performance molten salt.

### 2.2 Mechanism of Ion Conduction and Temperature Equilibrium

**FIGURE 2:**
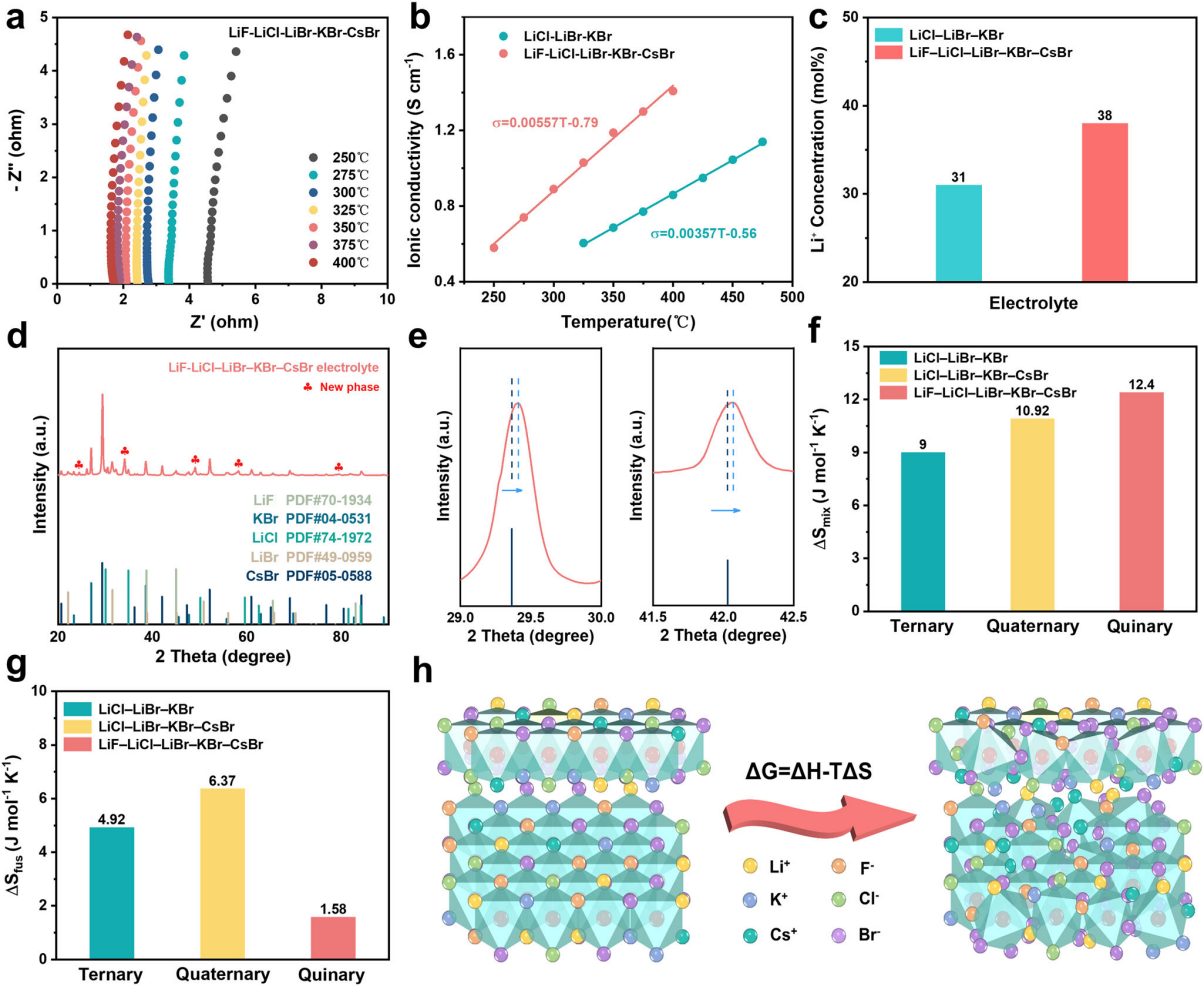
Ionic conduction, XRD characterization and high‐entropy regulation of LiF‐LiCl‐LiBr‐KBr‐CsBr molten salt. (a) Nyquist plots of LiF‐LiCl‐LiBr‐KBr‐CsBr at different temperatures. (b) The variation of ionic conductivity of LiCl‐LiBr‐KBr and LiF‐LiCl‐LiBr‐KBr‐CsBr with temperature. (c) Comparison of Li^+^ molar fraction between traditional LiCl‐LiBr‐KBr and LiF‐LiCl‐LiBr‐KBr‐CsBr molten salts. (d) XRD pattern of LiF‐LiCl‐LiBr‐KBr‐CsBr molten salt. (e) Enlarged partial view of Figure 2d. (f) Schematic diagram of lattice distortion caused by a multi‐component mixture. (g) Entropy of mixing (ΔS_mix_) and (h) entropy of fusion (ΔS_fus_) for ternary, quaternary, and quinary molten salt systems.

The ion transport kinetics of the molten salt were investigated using temperature‐dependent EIS. As shown in Figure 2a, the bulk resistance decreases significantly as the temperature rises from 250°C to 400°C, indicating the enhanced ion mobility of LiF‐LiCl‐LiBr‐KBr‐CsBr [41]. The conductivity‐temperature relationship (Figure 2b) shows that the quinary system consistently outperforms the ternary counterpart, reaching ∼1.19 S cm^−1^ at 350°C. This superior conductivity is fundamentally attributed to the strategic incorporation of LiF, which elevates the molar concentration of charge carriers (Li^+^) and entropy‐induced structural disorder. As shown in Figure 2c, the Li^+^ molar fraction increases from 31 mol% in the ternary system to 38 mol% in the quinary system, providing a higher density of mobile charge carriers. Meanwhile, the increase in entropy creates a more disordered local environment, in which Li^+^ ions move more freely and respond more rapidly to the applied electric field, further accelerating ion transport kinetics [20].

Beyond the superior ionic conductivity, the multi‐component design yields a remarkable reduction in the melting point, which is critical for broadening the operational temperature window of the molten salt. To elucidate the structural and thermodynamic origins of this phenomenon, a detailed analysis of the crystal lattice and entropy evolution has been conducted. XRD analysis (Figure 2d,e) reveals peak broadening and shifts, indicating the severe lattice distortion induced by multi‐component mixing [42]. This distortion weakens the binding forces between atoms within the lattice, thereby reducing the activation energy required for atomic diffusion [43, 44]. Consequently, the crystal structure can overcome these binding forces and transition to the liquid phase at a lower temperature. From a thermodynamic perspective, the entropy of mixing (ΔS_mix_) increases significantly with compositional complexity, stabilizing a disordered structure of the LiF‐LiCl‐LiBr‐KBr‐CsBr molten salt [45, 46]. As shown in Figure 2f, the ΔS_m__i__x_ of the quinary system increases significantly with the number of components reaching 12.4 J mol^−1^ K^−1^ for the quinary system, compared to 9 and 10.92 J mol^−1^ K^−1^ for ternary and quaternary systems, respectively, indicating that the solid phase of LiF‐LiCl‐LiBr‐KBr‐CsBr already exhibits a high degree of disorder due to multi‐component mixing. The entropy of fusion (ΔS_fus_) quantifies the increase in disorder when a substance melts, and can be determined from the DSC data. The ΔSfus value can be calculated using the following formula:

(1) $$\Delta S_{fus}=\frac{\Delta H_{fus}}{T_{m}}$$

where ΔH_fus_ is the enthalpy of fusion obtained by integrating the endothermic melting peak, and T_m_ is the corresponding melting temperature [47].

This drastically lower ΔS_fus_ (1.58 J mol^−1^ K^−1^) compared to the ternary (4.92 J mol^−1^ K^−1^) and quaternary (6.37 J mol^−1^ K^−1^) systems indicates an entropy pre‐saturation effect. The solid‐state quinary system has already achieved a high degree of structural disorder prior to melting. Because the solid phase is already in a highly disordered state (i.e., entropy pre‐saturation), it requires only a minimal additional entropy increment to transition into the liquid state. The schematic illustration in Figure 2h visually depicts the enhanced structural disorder, which aligns with the observed increase in configurational entropy and reduced phase transition entropy demand. Additionally, the multiple halide species in LiF‐LiCl‐LiBr‐KBr‐CsBr introduce complex local chemical environments and potential new phase interactions, as evidenced by additional diffraction features (Figure 2d). These factors further disrupt long‐range ordering and promote the formation of low‐energy eutectic configurations [48]. Collectively, lattice distortion, entropy enhancement, and phase evolution synergistically enable the simultaneous reduction of melting point and enhancement of ionic conductivity, rendering the quinary molten salt a promising candidate for advanced high‐temperature battery systems.

### 2.3 Design Principles of Low Melting Point Electrolytes

**FIGURE 3:**
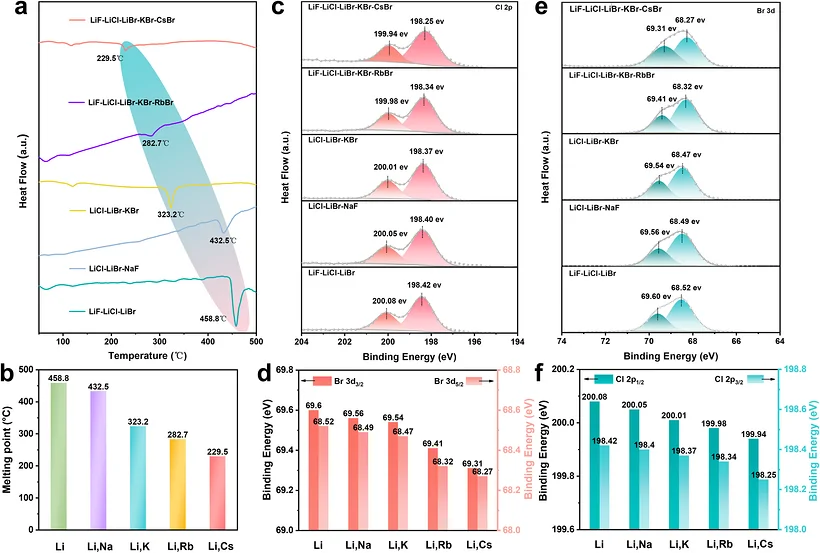
Thermal properties and XPS analysis of molten salt electrolytes. (a) DSC curves of different molten salts. (b) Melting points extracted from Figure 3a. (c) Fitted XPS Cl 2p spectra of different molten salts. (d) Cl 2p binding energies extracted from Figure 3c. (e) Fitted XPS Br 3d spectra of different molten salts. (f) Br 3d binding energies extracted from Figure 3e.

To further elucidate the relationship of structure and property, a series of cation‐substituted molten salts were systematically investigated (Table S2). As shown in Figure 3a,b, the all‐lithium LiF‐LiCl‐LiBr reference system exhibits the highest melting point at 458.8°C. Substituting a portion of Li^+^ with Na^+^ (LiCl‐LiBr‐NaF) yields an initial melting point decrease to 432.5°C. Further substitution with larger K^+^ (LiCl‐LiBr‐KF) reduces the melting point to 323.2°C. Incorporation of Rb^+^ (LiF‐LiCl‐LiBr‐KBr‐RbBr) and finally Cs^+^ (LiF‐LiCl‐LiBr‐KBr‐CsBr) leads to successive melting point reductions to 282.7°C and 229.5°C, respectively. The melting point decreases monotonically with increasing average cation radius, despite variations in exact salt composition and stoichiometry across the series.

To gain deeper insight into the electronic‐structure origin of this melting point trend, XPS analysis on the molten salts has been conducted. Figure 3c,e display the high‐resolution Cl 2p and Br 3d spectra, respectively, in which a corresponding shift of Cl 2p and Br 3d binding energies toward lower values with increasing cation size is observed, indicating weakened Coulombic interactions between cations and anions. This qualitative observation is further quantified in Figure 3d,f, where the monotonic decrease in binding energy with increasing cation size is explicitly plotted. According to the fundamental principle of XPS, the binding energy shifts of Cl 2p and Br 3d primarily reflect changes in their own chemical state, which in this system is predominantly governed by the polarizing power of the coordinated cations. It should be noted that the types and relative contents of halide anions also differ among the samples (e.g., some contain F^−^ while others do not). If variations in anion composition were the dominant factor, the binding energy shifts should either correlate with the F^−^ content or exhibit discontinuous jumps between F‐containing and F‐free samples. However, the data show a continuous, monotonic decrease regardless of the anionic composition. This indicates that the chemical environment of the halide anions is systematically altered by the introduction of larger, lower‐charge‐density cations, leading to a progressive weakening of their average Coulombic bonding strength with the surrounding cations.

According to the Empirical Electron Theory, the solid‐liquid phase transition of ionic crystals is primarily governed by the fracture of the strongest cation‐anion bonds that support the crystal lattice [49]. Mechanistically, the introduction of larger cations (Na^+^, K^+^, Rb^+^, Cs^+^) with lower charge densities creates heterogeneous bonding environments within the ionic lattice. In the all‐lithium (Li^+^) system, the small ionic radius (76 pm) and high charge density of Li^+^ result in a strong, homogeneous cation‐anion bonding network, high lattice energy, and, consequently, a high melting point. Upon introducing larger cations such as Na^+^ (95 pm) with lower charge density, the cation‐anion interactions are weakened relative to those of Li^+^, effectively incorporating weaker bonding sites into the originally uniform strong‐bonding network. By analogy, the larger the radius of the introduced cation (e.g., K^+^: 138 pm, Rb^+^: 152 pm, Cs^+^: 167 pm), the lower its charge density and the weaker its interaction with the anions. In the quinary LiF‐LiCl‐LiBr‐KBr‐CsBr system, three bonding modes coexist, including strong interaction (Li^+^‐anion), moderate interaction (K^+^‐anion), and weak interaction (Cs^+^‐anion). The coexistence of these distinct bond‐strength inhomogeneities disrupts long‐range lattice order. As a result, the lattice becomes more susceptible to disruption by thermal motion at lower temperatures, enabling a further significant reduction in the melting point.

In these systems, the anions (Cl^−^ and Br^−^) serve as a relatively fixed chemical background, while the cation species (Li^+^, Na^+^, K^+^, Rb^+^, Cs^+^) act as the primary variable that governs the cation‐anion bonding strength under this fixed anionic environment. From these findings, a general design rule for low‐melting‐point alkali metal halide molten salts can be established: the incorporation of large‐radius, low‐charge‐density cations effectively depresses the melting point in a systematic manner. This compositional modulation induces bond‐strength inhomogeneity, disrupts long‐range lattice ordering, and lowers the overall lattice energy. Collectively, these effects provide a clear and rational pathway for designing low‐melting‐point molten salts for advanced electrochemical applications.

### 2.4 Performance Validation of Single Thermal Battery

In the following, the practical performance of the high‐entropy molten salt was evaluated in thermal batteries.

**FIGURE 4:**
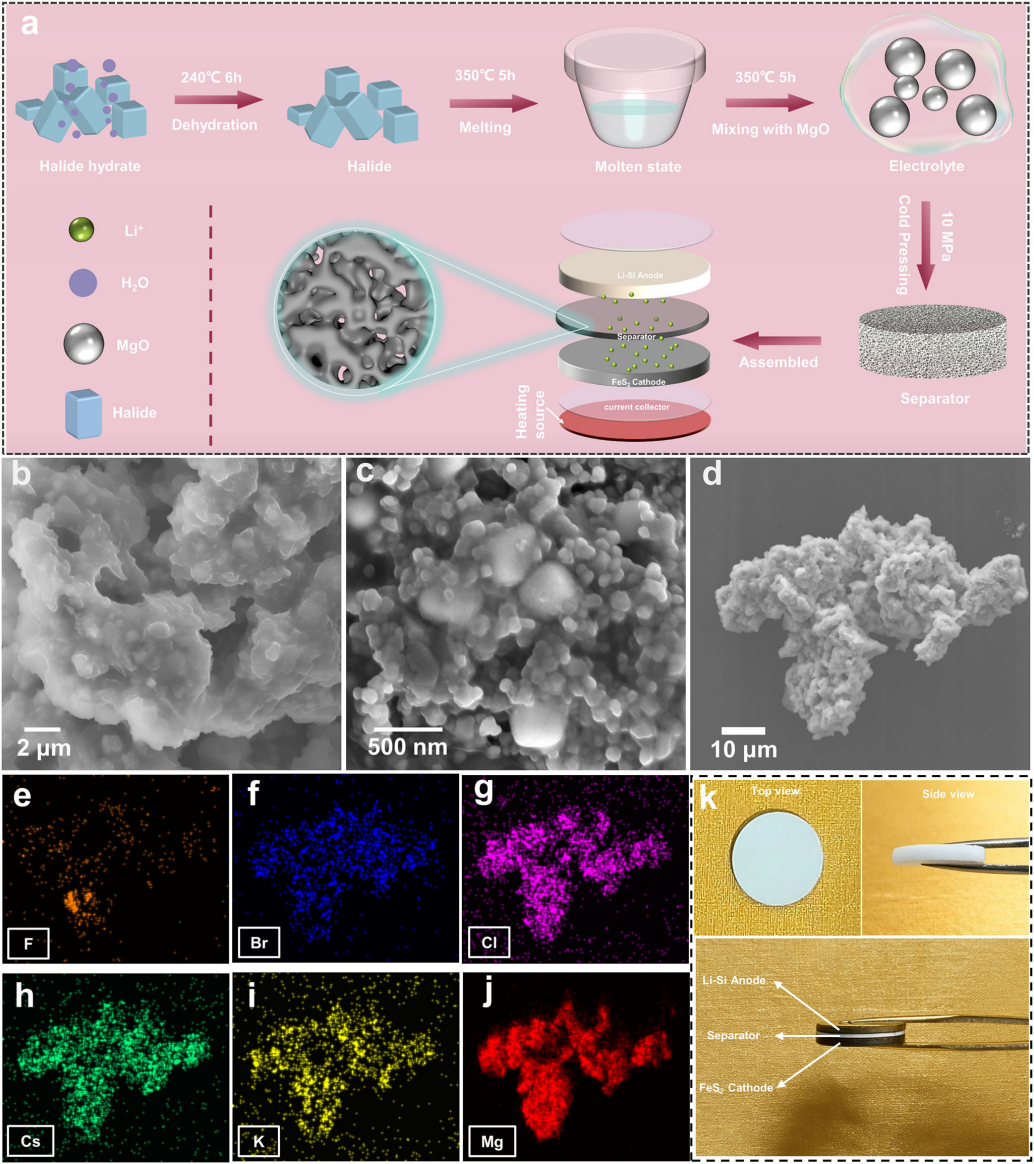
Preparation, morphology, and elemental distribution of LiF‐LiCl‐LiBr‐KBr‐CsBr electrolyte. Schematic illustration of the preparation of LiF‐LiCl‐LiBr‐KBr‐CsBr electrolyte and its application as a separator in thermal batteries. SEM images (b, c) of LiF‐LiCl‐LiBr‐KBr‐CsBr and corresponding elemental mappings (d‐j) of Br, Cl, Cs, K, and Mg of LiF‐LiCl‐LiBr‐KBr‐CsBr. (k) Optical images of the electrolyte pellet in top and side views (Top), Optical images of the corresponding assembled battery (Bottom).

The preparation process is shown in Figure 4a. Given the hygroscopic nature of alkali metal halides, the precursors were pre‐dried at 240°C for 6 h to remove residual moisture. The salts were then thoroughly ground in an agate mortar for 2 h inside an argon glove box (H_2_O and O_2_ < 0.1 ppm) to realize macroscopically homogeneous mixing. The uniformity of the ground powder was verified by SEM‐EDS elemental mapping (Figure S3), which revealed uniform elemental distributions without local segregation. Subsequently, the mixture was melted at 350°C to ensure compositional homogeneity. Furthermore, DSC analysis (Figure 1g) exhibited only a single endothermic melting peak, confirming the formation of a homogeneous eutectic molten salt system rather than a mechanical mixture of individual salts. During thermal battery operation, the molten salt exhibits extremely high fluidity after melting at high temperatures, and the device is subjected to significant mechanical stresses such as acceleration, shock, rotation, and vibration. Therefore, the electrolyte must be securely fixed using a binder. Here, MgO is selected as the binding filler at a loading of 50 wt.% (based on the total mass of the composite electrolyte) to improve the stability of the electrolyte. The resulting material was then pressed into dense pellets (16 mm in diameter) for device fabrication. The morphology of the electrolyte was analyzed using scanning electron microscopy (SEM). As shown in Figure 4b–d and Figure S4, the molten salt uniformly coats the surface of the MgO particles, forming a continuous and dense matrix that effectively reduces interparticle boundaries and enhances structural compactness. The corresponding elemental mappings (Figure 4e–j) reveal a homogeneous distribution of Br, Cl, Cs, K, and Mg, indicating intimate mixing of the molten salt and MgO across multiple length scales. The relatively weak F signal is attributed to its lower content in the composition. Based on the as‐prepared electrolyte, a single thermal battery was assembled. The optical image shown in Figure 4k confirms that this electrolyte has excellent formability and the relative position of positive electrode, electrolyte, and negative electrode in the monomer battery. As shown in Figure S5, a comparison of the electrolyte's ionic conductivity with and without MgO reveals a decrease in the macroscopic ionic conductivity of the system. This is because MgO itself is an electrical insulator. Its particles are uniformly dispersed in the molten salt, disrupting the originally continuous ion migration pathways, increasing the tortuosity of the pores within the separator, and prolonging the ion migration paths, ultimately leading to a marked reduction in the overall conductivity.

**FIGURE 5:**
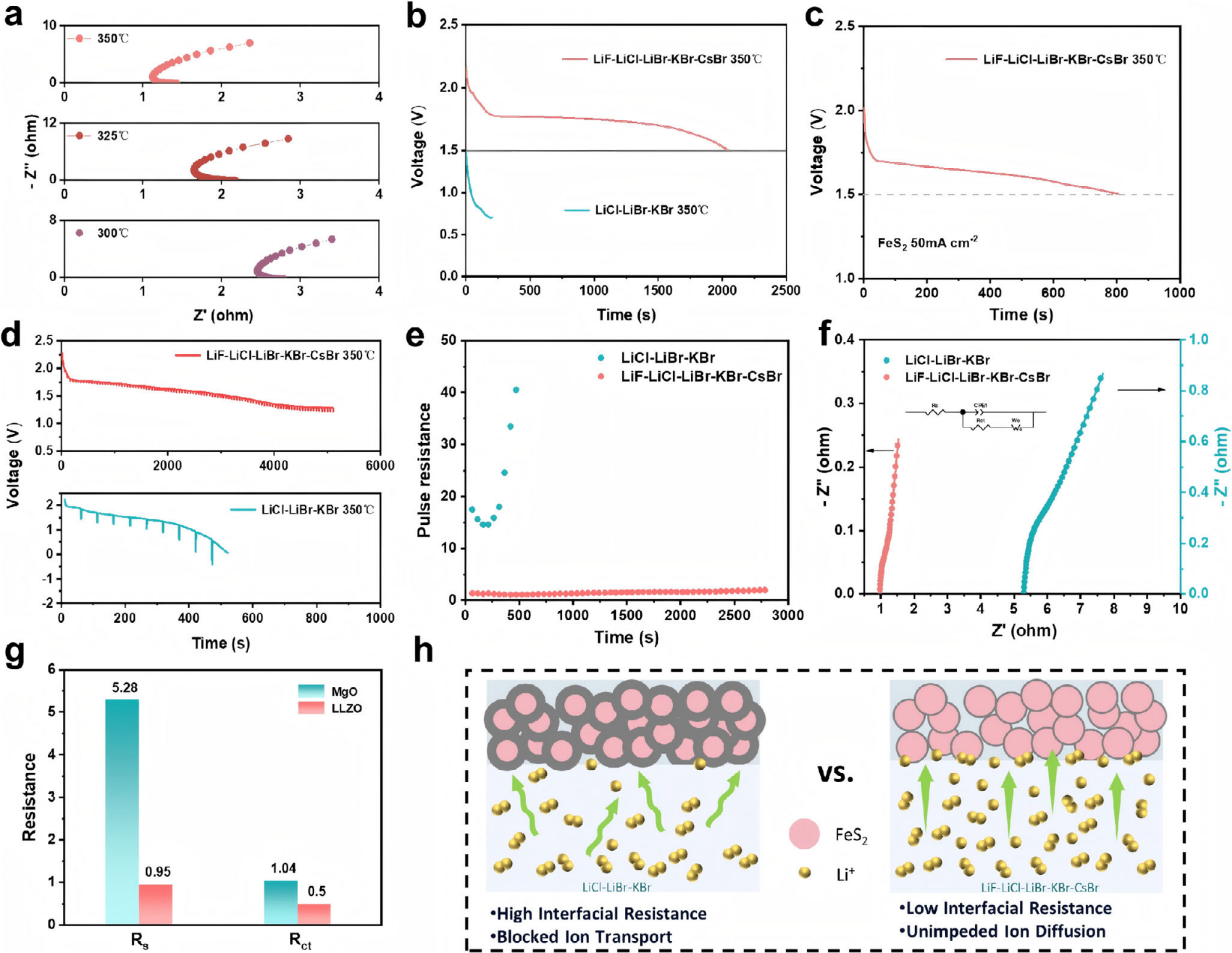
Electrochemical performance and ion transport behavior of molten salt electrolytes. (a) Nyquist plots of LiF‐LiCl‐LiBr‐KBr‐CsBr at 300°C, 325°C, and 350°C. (b) Discharge curves of LiCl‐LiBr‐KBr and LiF‐LiCl‐LiBr‐KBr‐CsBr at a current density of 25 mA cm^−2^. (c) Discharge curve of LiF‐LiCl‐LiBr‐KBr‐CsBr at a current density of 50 mA cm^−2^. (d) Pulse discharge (12.5 mA cm^−2^, 50 s to 25 mA cm^−2^, 1 s) curves of LiCl‐LiBr‐KBr and LiF‐LiCl‐LiBr‐KBr‐CsBr. (e) Pulse resistance of LiCl‐LiBr‐KBr and LiF‐LiCl‐LiBr‐KBr‐CsBr. (f) EIS spectra of LiCl‐LiBr‐KBr and LiF‐LiCl‐LiBr‐KBr‐CsBr during the discharge process at 350°C. The inset shows the equivalent circuit of the electrolytes. (g) Comparison of EIS‐fitted resistances (R_s_ and R_ct_) for the LiCl‐LiBr‐KBr and LiF‐LiCl‐LiBr‐KBr‐CsBr electrolytes. (h) Schematic illustration of ion transport behavior.

The electrochemical performance of the thermal batteries was systematically evaluated by EIS and discharge measurements. As shown in Figure S6, the LiCl‐LiBr‐KBr electrolyte exhibits extremely high impedance at 300°C due to the absence of a continuous ionic conduction pathway below its melting point. Although partial melting occurs at 325°C, insufficient liquid‐phase formation still results in high resistance. Even at 350°C, the impedance remains as high as ∼21 Ω, indicating sluggish ion transport. In contrast, the LiF‐LiCl‐LiBr‐KBr‐CsBr electrolyte, due to its much lower melting point (229.5°C), exhibits a significantly reduced impedance of 2.8 Ω at 300°C, which further decreases to 1.46 Ω at 350°C (Figure 5a), demonstrating the markedly enhanced ionic transport capability. Figure 5b–d and Figure S7 show the discharge test results of the two electrolytes at different temperatures. At 300°C, the LiCl‐LiBr‐KBr cell fails to sustain discharge, whereas the quinary electrolyte enables stable operation, albeit without a pronounced voltage plateau due to the limited ion mobility at relatively low temperature. At 325°C, the LiCl‐LiBr‐KBr system still suffers from insufficient melting and high resistance, resulting in discharge voltages below 1 V. In contrast, the LiF‐LiCl‐LiBr‐KBr‐CsBr electrolyte can sustain discharge for 325 s under a cutoff voltage of 1.5 V. When the temperature reaches 350°C, it maintains a stable voltage plateau of approximately 1.72 V at a current density of 25 mA cm^−2^, with a prolonged discharge duration of up to 2400 s and a specific capacity of 266 mAh g^−1^ at a cutoff voltage of 1.5 V (Figure 5b). Under a higher current (0.1 A), the cell still maintains a discharge time of 807 s, corresponding to a specific capacity of 179 mAh g^−1^ (Figure 5c).

Pulse discharge tests further highlight the improved electrochemical kinetics (Figure 5d) of the quinary electrolyte. The LiF‐LiCl‐LiBr‐KBr‐CsBr system exhibits higher initial pulse voltage (∼2.28 V) and a higher steady‐state plateau (∼1.73 V) compared to the LiCl‐LiBr‐KBr system (∼2.24 and ∼1.55 V, respectively). More importantly, the quinary electrolyte shows significantly reduced voltage decay, maintaining ∼1.5 V after 3147 s, whereas the ternary system drops below this value within 214 s. This improved performance can be attributed to the lower melting point and enhanced fluidity of the quinary electrolyte, which facilitate better electrode‐electrolyte contact and faster Li^+^ transport, thereby suppressing polarization of the battery.

The superior performance of the quinary electrolyte can also be supported by the evolution of internal resistance during discharge. Both systems exhibit an initial decrease followed by an increase in resistance. The resistance of LiCl‐LiBr‐KBr decreases from an initial 17.5 to 14.5 Ω and then sharply rises to 40.5 Ω, whereas the LiF‐LiCl‐LiBr‐KBr‐CsBr system shows a much smaller variation, decreasing from 1.29 to 1.05 Ω and then gradually increasing to 1.91 Ω. The initial decrease is associated with the formation of metallic iron atoms (e.g., Fe) [50], while the subsequent increase originates from polarization buildup during discharge. The slower resistance rise in the LiF‐LiCl‐LiBr‐KBr‐CsBr cell indicates suppressed polarization and enhanced structural stability, consistent with its superior electrochemical performance [51].

The total polarization (*R*) of a single battery can be calculated by the following equation:

(2) $$R=\frac{V_{c}-V_{p}}{I_{p}-I_{c}}$$

where *V_c_* and *V_p_* are the instantaneous voltages immediately before and after the application of the pulse current, respectively, and *I_p_* and *I_c_* are the the pulse current and working current.

As shown in Figure 5e, the LiCl‐LiBr‐KBr system exhibits a high internal resistance exceeding 14 Ω, which accounts for its rapid voltage decay. In comparison, the LiF‐LiCl‐LiBr‐KBr‐CsBr system shows a much lower average resistance of about 1.45 Ω, indicating substantially improved electrochemical kinetics.

To further demonstrate the superior ion transport kinetics of the LiF‐LiCl‐LiBr‐KBr‐CsBr electrolyte in thermal batteries, EIS and the corresponding interfacial mechanisms have been analyzed. Figure 5f presents the Nyquist plots of symmetric cells with LiCl‐LiBr‐KBr and LiF‐LiCl‐LiBr‐KBr‐CsBr electrolytes at 350°C, along with the corresponding equivalent circuit model (inset of Figure 5f). Both systems display a well‐defined semicircle in the high‐to‐medium frequency region, associated with the interfacial charge transfer process, followed by a linear Warburg tail in the low‐frequency region corresponding to Li^+^ diffusion [52, 53]. The diameter of the semicircle for the LiF‐LiCl‐LiBr‐KBr‐CsBr cell is notably smaller than that of the LiCl‐LiBr‐KBr cell, suggesting a reduced charge transfer resistance [54, 55, 56].

After fitting the EIS data using ZView software, the quantitative resistance parameters (Figure 5g) reveal that the LiF‐LiCl‐LiBr‐KBr‐CsBr system possesses markedly lower resistive components, with ohmic resistance (*R_s_*) and charge transfer resistance (*R_ct_*) values of 0.95 and 0.50 Ω, respectively, compared to 5.28 and 1.04 Ω for the LiCl‐LiBr‐KBr cell. These results confirm that the high‐entropy electrolyte effectively suppresses both ohmic and interfacial resistances, enabling rapid ion transport and improved high‐rate performance in thermal batteries.

The underlying ion transport mechanism is schematically illustrated in Figure 5h. The LiCl‐LiBr‐KBr electrolyte forms a resistive and tortuous interface with the FeS_2_ cathode, which hinders Li^+^ migration. In stark contrast, the LiF‐LiCl‐LiBr‐KBr‐CsBr electrolyte forms a more compatible and conductive interface with FeS_2_, enabling low interfacial resistance and unimpeded Li^+^ diffusion. This improved interfacial compatibility arises from the synergistic effect of LiF and CsBr, which optimizes the electrolyte‐electrode interfacial structure and promotes rapid Li^+^ migration, consistent with the reduced impedance values observed in Figure 5f,g.

### 2.5 Performance Evaluation of Thermal Battery Prototype

**FIGURE 6:**
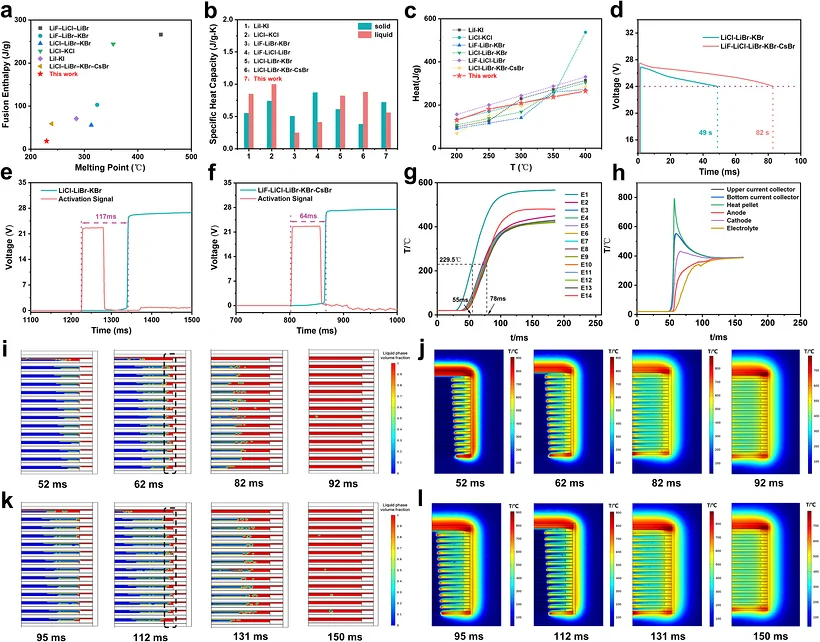
Thermal properties, energy consumption, and thermal battery prototype performance. (a) Comparison of melting points and fusion enthalpies of this work with other commonly used electrolytes. (b) Comparison of the solid‐ and liquid‐state specific heat capacities measured in this work with those of other electrolytes in the literature. (c) Comparison of the energy consumption of the LiF‐LiCl‐LiBr‐KBr‐CsBr electrolyte with that of other electrolytes at different temperatures. (d) Galvanostatic discharge performance of a thermal battery prototype consisting of 14 serially connected single cells at a current density of 25 mA cm^−2^. (e,f) The activation time of the thermal battery prototype. (g) Average temperature rising curves of the LiF‐LiCl‐LiBr‐KBr‐CsBr electrolytes. (h) Temperature curves of domain point probes of the LiF‐LiCl‐LiBr‐KBr‐CsBr battery. (i) Phase‐change cloud diagram of LiF‐LiCl‐LiBr‐KBr‐CsBr battery at different times. (j) Temperature distribution cloud of LiF‐LiCl‐LiBr‐KBr‐CsBr battery at different times. (k) Phase‐change cloud diagram of LiCl‐LiBr‐KBr battery at different times. (l) Temperature distribution cloud of LiCl‐LiBr‐KBr battery at different times.

Having validated the electrochemical kinetics at the single‐cell level, the engineering applicability of the electrolyte at the device level has been evaluated, with particular emphasis on thermal response and system‐level performance. Based on the melting enthalpy data shown in Figure S2, Figure 6a compares the melting point and enthalpy of fusion of the electrolyte developed in this work with those of other commonly used thermal battery electrolytes [57]. It can be clearly seen that the LiF‐LiCl‐LiBr‐KBr‐CsBr electrolyte exhibits both the lowest melting point and the lowest enthalpy of fusion among representative thermal battery electrolytes. These characteristics are highly favorable for rapid thermal activation and reduced energy consumption. Figure 6b further compares the specific heat capacities of this electrolyte in both solid and liquid states [57]. The relatively low specific heat capacity indicates reduced sensible heat requirements during the heating process, which contributes to the accelerated thermal response. To quantitatively evaluate and compare the thermal loads of different electrolytes, the total heat input required to raise the electrolyte from room temperature to the operating temperature was calculated under adiabatic conditions according to the following equation:

(3) $$H_{\mathrm{sum}}=(T_{f}-T_{a})\cdot C_{p}(\mathrm{cr}.)+\Delta H_{\mathrm{fusion}}+(T_{b}-T_{f})\cdot C_{p}(\mathrm{liq}.)$$

where *H*_sum_ is the total heat required to raise the temperature of the electrolyte in a thermal battery from room temperature to its operating temperature, *T_f_* is the melting point, *T_a_* is the initial (room) temperature, *C_p_* (cr.) is the specific heat capacity in the solid state, Δ*H*_fusion_ is the enthalpy of fusion, *T_b_* is the battery operating temperature, and *C_p_* (liq.) is the specific heat capacity in the liquid state.

As summarized in Figure 6c, it can be clearly observed that when the target operating temperature is set at 350°C, the LiF‐LiCl‐LiBr‐KBr‐CsBr electrolyte exhibits the lowest total heat requirement (354 J g^−1^), arising from its low melting point, low fusion enthalpy, and reduced heat capacities. This reduced thermal load directly translates into lower heating‐agent demand during activation, enabling reductions in system weight and volume and thereby improving both gravimetric and volumetric energy densities. Such advantages are particularly important for applications requiring lightweight and high‐specific‐energy power sources.

The practical implications of these thermodynamic advantages were verified in a prototype battery consisting of 14 single cells connected in series. As shown in Figure 6d, under a constant current density of 25 mA cm^−2^, the prototype utilizing the LiF‐LiCl‐LiBr‐KBr‐CsBr electrolyte delivers a stable voltage plateau (>24 V) for 82 s, significantly outperforming the 49 s duration of the conventional LiCl‐LiBr‐KBr system. Beyond discharge capacity, activation speed is a critical metric for thermal batteries. As shown in Figure 6e,f, the quinary electrolyte enables ultrafast activation within 64 ms, compared to 117 ms for the ternary system. To further investigate the origin of the rapid activation, a thermal activation simulation has been established (Figures S8 and S9). The temperature evolution (Figure 6g,h) reveals pronounced spatial heterogeneity, with melting initiation occurring at different locations within 55–78 ms. Notably, the experimentally observed activation time (64 ms) correlates closely with the melting of electrolyte regions near the heat source, rather than the average melting behavior of the entire system [58].

This spatiotemporal heterogeneity is further visualized by the phase‐change and temperature distribution cloud diagrams in Figure 6i–l. Simulation results indicate that the LiF‐LiCl‐LiBr‐KBr‐CsBr electrolyte particles near the igniter begin to melt at 62 ms (Figures 6i and 6j), significantly earlier than the LiCl‐LiBr‐KBr system (112 ms, Figures 6k and 6l). These results confirm that macroscopic voltage generation does not require the complete electrolyte melting but instead relies on the formation of a continuous, low‐resistance conductive pathway near the heat source, as shown in Figure S10. In addition, the relatively slow temperature rise of the electrolyte compared to the anode highlights that the thermal inertia of the electrolyte itself is the primary bottleneck for activation (Figure 6h). The reduced melting point and thermal load of the LiF‐LiCl‐LiBr‐KBr‐CsBr electrolyte effectively mitigate the limitation. Overall, the combination of low melting point, reduced thermal load, fast activation, and stable discharge performance establishes the LiF‐LiCl‐LiBr‐KBr‐CsBr electrolyte as a promising candidate for high‐performance thermal batteries, particularly in applications demanding rapid response and high energy density.

## 3 Discussion

In conclusion, by precisely adjusting the incorporation of CsBr and LiF, the intrinsic trade‐off between the low melting point and high ionic conductivity in molten salt electrolytes has been effectively overcome. A high‐entropy quinary LiF‐LiCl‐LiBr‐KBr‐CsBr electrolyte with superior performance has been designed and synthesized, in which multi‐component mixing induces pronounced lattice distortion and elevated configurational entropy, synergistically enabling both melting‐point depression and enhanced ion transport. The optimized electrolyte exhibits an ultralow melting point of 229.5°C, a high ionic conductivity of up to 1.19 S cm^−1^ at 350°C, and a low total thermal load of only 354 J g^−1^. When implemented in the thermal battery single cell, it delivers a stable discharge plateau of approximately 1.72 V with a specific capacity reaching 266 mAh g^−1^ at 350°C. Furthermore, a prototype thermal battery stack comprising 14 cells further demonstrates ultrafast activation within 64 ms and sustained discharge for 82 s at a current density of 25 mA cm^−^^2^ (with a cutoff voltage of 24 V), significantly outperforming conventional LiCl‐LiBr‐KBr systems. Multiphysics simulations reveal that such millisecond‐level ultrafast activation in the high‐entropy electrolyte originates from the rapid formation of a critical, percolated ionic conduction pathway, rather than complete electrolyte melting, thereby challenging the conventional temperature‐based activation criterion. Overall, this work establishes high‐entropy engineering as a powerful design paradigm for decoupling key physicochemical properties in molten salts, providing new opportunities for the development of next‐generation high‐power, fast‐response thermal batteries and other high‐temperature electrochemical systems.

## 4 Materials and Methods

### 4.1 Chemicals

Lithium fluoride (LiF, 99%), lithium chloride (LiCl, 99%), lithium bromide (LiBr, 99%), potassium bromide (KBr, 99%), and magnesium oxide (MgO, 99%) were obtained from Macklin (Shanghai, China). Cesium bromide (CsBr) was purchased from Aladdin (Shanghai, China). Lithium silicon alloy (LiSi) was obtained from Tianjin Zhongneng Lithium Co., Ltd. All chemical reagents in this work were of analytical grade and used without further purification. Deionized water was used for all experimental procedures.

### 4.2 Characterizations

X‐ray diffraction (XRD) measurements were conducted on a D8 Bruker X‐ray diffractometer with Cu Kα radiation (λ = 1.5406 Å) as the radiation source. Scanning electron microscopy (SEM) was carried out on an SEM Hitachi S‐8010 microscope equipped with an energy‐dispersed X‐ray spectroscope (EDS) detector (Oxford X‐ray Max20, Oxford Instruments plc, Oxford, UK). Thermogravimetric analysis (TG) and differential scanning calorimetry (DSC) were performed on a Diamond instrument (STA 449, PerkinElmer, USA) over a temperature range of 50°C–500°C at a heating rate of 10°C min^−1^ in an Ar atmosphere. X‐ray photoelectron spectroscopy (XPS) was measured on a Thermo Scientific K‐Alpha.

### 4.3 Electrochemical Measurements

All battery assembly procedures were conducted within an argon‐filled glove box equipped with a continuous gas purification system (MB‐200B‐MOD, M. Braun, Germany). The levels of H_2_O and O_2_ within the atmosphere were strictly maintained below 0.1 ppm. The single cell consists of a cathode, an electrolyte, and an anode. The cathode was fabricated with 50 wt.% FeS_2_ and 50 wt.% electrolyte. The electrolyte was composed of 50 wt.% LiF‐LiCl‐LiBr‐KBr‐CsBr molten salt and 50 wt.% MgO. MgO is electrochemically inert and does not participate in ion transport, and the ionic conductivity originates exclusively from the molten salt phase. The anode was prepared using commercial Li‐Si (Li 44 wt.%‐Si 56 wt.%). All of them were pressed into 16 mm pellets under 12 MPa pressure. The masses of the cathode, the electrolyte, and the anode within every single cell were set to 0.25, 0.35, and 0.15 g, respectively.

The thermal battery prototype is constructed by connecting 14 single cells in series to deliver the required operating voltage. Given the plateau voltage of approximately 1.72 V per single cell, the 14‐cell series configuration yields an output voltage of approximately 24 V (14 × 1.72 V ≈ 24.08 V), which is set as the effective operating voltage threshold. Terminal heating layers are incorporated at both ends of the stacks, each composed of multiple asbestos sheets and an integrated heating strip. The asbestos sheets serve as electrical insulation, while the heating strips facilitate rapid temperature rise during startup to initiate the electrochemical reactions. An ignition strip is mounted externally on the stack to provide the necessary thermal energy in the initial phase, thereby activating the internal chemical processes. The entire assembly is enclosed within an insulation layer, which prevents electrical short circuits and shields the system from external environmental conditions. A thermal insulation layer is further applied around the stack to minimize heat dissipation and maintain an optimal operating temperature, which is critical for sustaining battery performance. Finally, the battery cover and casing are joined and hermetically sealed by welding, ensuring structural integrity and a leak‐tight enclosure.

The electrochemical properties of the thermal batteries were studied with an Autolab electrochemical workstation (Metrohm, PGSTAT302N, Switzerland). The assembled cells were connected to the electrochemical workstation and inserted into an Ar‐filled tube furnace (ROTH 75/200/18, Germany) and left at 350°C for 20 min before each measurement. Galvanostatic charge/discharge tests were measured at 25 and 50 mA cm^−2^. The pulse discharge (PD) measurements were performed at a short current pulse of 25 mA cm^−2^ for 1 s every 50 s during the discharge at 12.5 mA cm^−2^. The electrochemical impedance spectroscopy (EIS) measurements were conducted within the frequency range of 1 to 10 MHz at a certain voltage. The ionic conductivity value can be calculated using the following formula:

(4) $$\sigma =\frac{L}{R\cdot S}$$

where σ, L, R, and S are electrolyte conductivity, pellet thickness, material resistance, and material cross‐sectional area, respectively.

### 4.4 Finite Element Simulation of Transient Thermal Transfer Effect

The transient thermal transfer simulation model for the thermal battery activation process was developed using the COMSOL Multiphysics coupling software platform. Its core governing equation is as follows:

(5) $$\rho C_{p}\frac{\partial T}{\partial t}=k\nabla \left(\nabla T\right)+Q_{s}+Q_{c}-Q_{m}$$

where *ρ*, *C_p_*, and *k* represent the material density, specific heat capacity, and thermal conductivity, respectively; *T* is the temperature; *t* is time. *Q_s_* denotes the heat released by the pyrotechnic system, such as the heating sheets and the igniter strip. *Q_m_* accounts for the latent heat absorbed during the melting phase change of the electrolyte (primarily located in the separator and minimally in the cathode sheet). *Q_c_* represents the heat loss due to convective heat transfer between the battery casing and the external environment, calculated by:

(6) $$Q_{c}=h\left(T_{e}-T_{w}\right)$$

Here, *h* is the convective heat transfer coefficient, while *T*_e_ and *T_w_* ​ are the ambient air temperature and the battery outer surface temperature, respectively. *Q_m_* accounts for the latent heat absorbed during the melting phase change of the electrolyte (primarily located in the separator and minimally in the cathode sheet):

(7) $$Q_{m}=\rho \alpha \Delta H_{m}\frac{\partial \theta }{\partial t}$$

In this equation, α is the mass fraction of the electrolyte within the phase change material, Δ*H_m_* is the melting heat per unit mass of the electrolyte, and θ is the liquid phase volume fraction of the electrolyte.

The model utilizes a 2D axisymmetric geometry to balance computational efficiency and accuracy. The heat sources (igniter strip and heating sheets) were treated as moving heat sources, with their heat power density accurately described by user‐defined functions. Taking the igniter strip as an example, its thermal power density *Q_i_* is expressed as:

(8) $$Q_{i}=\frac{Q_{v}}{a/v_{i}}\cdot f\left(v_{i}t-x\right)$$

where *Q_v_* is the Heat density of the ignition strip, a is the width of the combustion reaction zone, and vi​ is the burning rate. *f*(*u*) is a piecewise function determining the spatial and temporal distribution of the heat source:

(9) $$f\left(u\right)=\left\{\begin{array}{c} 1,0\;<\;u\;\leq \;a\\ 0,U\leq 0\vee u>a \end{array}\right.$$

The cathode and separator sheets in the model were defined as phase change domains, with the phase change temperature set to the melting point of the electrolyte. The phase change heat was adjusted based on the actual electrolyte content. The thermal physical parameters of all the components required in the model are shown in the following Table S3.

## Author Contributions

Conceptualization: B.S., T.Q.; Methodology: B.S., G.W., T.Q.; Investigation: B.S., T.S., F.Y., Y.Z., Visualization: B.S., Supervision: Y.Zh., T.Q., Writing – original draft: B.S., Writing – review & editing: B.S., G.W., T.Q., Y.Zh.

## Conflicts of Interest

The authors declare no conflicts of interest.

## Supporting information




**Supporting file**: advs76847‐sup‐0001‐SuppMat.docx

## Data Availability

The authors have nothing to report.
