# Supplementary material for: Unlocking Low‐Melting‐Point and High‐Conductivity Molten Salts via High‐Entropy Strategy with Demonstration in Thermal Batteries
Source: Adv Sci (Weinh). 2026 Sep 3;13(59):e76847. doi: 10.1002/advs.76847 (PMC13540152; doi:10.1002/advs.76847)
Supplement: Supplementary file 1 [file ADVS-13-e76847-s001.docx]

**Unlocking Low-Melting-Point and High-Conductivity Molten Salts via High-Entropy Strategy with Demonstration in Thermal Batteries**

Binchao Shi ^a^, Ting Sun ^b^, Fushan Yu ^b^, Yi Zhang ^b^, Gaowu Wang ^c^*, Ting Quan ^a^*, and Yan-li Zhu ^a^*

*^a^ State Key Laboratory of Explosion Science and Safety Protection, Beijing Institute of Technology, Beijing 100081, China*

*^b^ Xi’an North Qinghua Electromechanical Co., Ltd., Xi’an 710025, China*

*^c^ BYD Lithium Battery Co.,Ltd., Shenzhen 518000, China*

^*^Corresponding author.

E-mail: wang.gaowu@fdbatt.com (Gaowu Wang);

[6120210087@bit.edu.cn (Ting Quan);](mailto:6120210087@bit.edu.cn%20(Ting%20Quan);)

[zhuyanli1999@bit.edu.cn](mailto:zhuyanli1999@bit.edu.cn) (Yanli Zhu)

**Figure S1. DSC curves of the molten salts with different molar compositions.** The detailed molar ratios of each sample are listed in Table 1. Samples No. 5 and No. 12 are presented in Figure 1c and Figure 1g, respectively.

**Note S1.**

DSC measurements were performed to screen molten‑salt compositions with low melting points. The typical samples selected for subsequent electrochemical tests correspond to No. 5 and No. 12, as shown in the main text.

**Figure S2. DSC and TG** **curves**. (a) LiCl-LiBr-KBr (25-37-38 mol%), (b) LiF-LiCl-LiBr-KBr-CsBr (13-25-37-12-13 mol%). The shaded areas represent the melting endotherms, indicating the melting enthalpies.

**Note S2.**

Combined DSC‑TG characterization quantifies melting enthalpies and thermal stability of two typical electrolytes, confirming the advantage of the quinary high‑entropy molten salt.

**Figure S3. SEM and corresponding EDS elemental mapping of F, Cl, Br, K and Cs for LiF, LiCl, LiBr, KBr, CsBr mixed salt after sufficient grinding.**

**Note S3.**

SEM and EDS elemental mapping characterizations are carried out to verify the mixing uniformity of the five-component molten salt mixture. Homogeneous distribution of all constituent elements without local segregation is observed in microscale regions, which confirms that sufficient grinding achieves uniform blending of LiF, LiCl, LiBr, KBr, CsBr powders.

**Figure S4. SEM images of MgO at different scales**.

**Note S4.**

SEM observations reveal the micromorphology of MgO powder used as raw material for molten‑salt electrolytes, providing intuitive structural information for electrolyte preparation.

**Figure S5. Ionic conductivity of molten salt electrolytes with and without MgO binder. (a) Quinary LiF-LiCl-LiBr-KBr-CsBr electrolyte; (b) Ternary LiCl-LiBr-KBr electrolyte.**

**Note S5.**

Figure S5 compares the ionic conductivity of quinary LiF-LiCl-LiBr-KBr-CsBr molten salt electrolyte and ternary LiCl-LiBr-KBr molten salt electrolyte before and after adding 50 wt% MgO binder. The test results quantitatively demonstrate that the incorporation of insulating MgO filler significantly reduces the ionic conductivity of both molten salt systems, which originates from the increased tortuosity of ion migration channels induced by uniformly dispersed MgO particles. These results illustrate the trade-off between mechanical structural stability and ionic conductivity brought by MgO binder in molten salt electrolyte design for thermal batteries.

**Figure S6. Nyquist plots of LiCl-LiBr-KBr at 300 °C, 325 °C, and 350 °C.**

**Note S6.**

Variable‑temperature impedance tests of the ternary LiCl‑LiBr‑KBr molten salt were carried out to compare its ionic conduction behavior with the quinary high‑entropy system in the main text.

**Figure S7. Discharge curves of LiCl-LiBr-KBr and LiF-LiCl-LiBr-KBr-CsBr at a current density of 25 mA cm^-2^.**

**Note S7.**

Galvanostatic discharge tests at 25 mA cm^-^² directly verify that the quinary molten‑salt electrolyte delivers superior discharge performance relative to the traditional ternary electrolyte.

**Figure S8. Mesh model of the simulated thermal battery prototype.**

**Note S8.**

Finite element mesh generation was performed for thermal‑field simulation of the thermal battery.

**Figure S9.** **The geometric model of the thermal battery and the position of the domain point probe (colorful dots represent the location of the probe).

Note S9.**

The finite‑element geometric model of the serially‑connected thermal battery stack was established, and domain‑point probes were set inside the electrolyte to record real‑time temperature variation during discharge simulation, where the ignition strip burns from top to bottom.

**Figure S10. Schematic of conductive pathway evolution driven by electrolyte melting.**

**Note S10.**

This schematic illustrates the stepwise formation of continuous ion‑conductive channels with temperature rise. White regions represent unmelted solid electrolyte; molten salt gradually aggregates and connects to form conductive pathways, thereby activating the thermal battery.

**Table S1.** Molar Compositions (mol%) of the Studied Halide Systems

| **No.** | **LiF** | **LiCl** | **LiBr** | **KBr** | **CsBr** |
| --- | --- | --- | --- | --- | --- |
| 1 | 0 | 25 | 37 | 33 | 5 |
| 2 | 0 | 25 | 37 | 28 | 10 |
| 3 | 0 | 25 | 37 | 27 | 11 |
| 4 | 0 | 25 | 37 | 26 | 12 |
| 5 | 0 | 25 | 37 | 25 | 13 |
| 6 | 0 | 25 | 37 | 24 | 14 |
| 7 | 0 | 25 | 37 | 23 | 15 |
| 8 | 5 | 25 | 37 | 20 | 13 |
| 9 | 10 | 25 | 37 | 15 | 13 |
| 10 | 11 | 25 | 37 | 14 | 13 |
| 11 | 12 | 25 | 37 | 13 | 13 |
| 12 | 13 | 25 | 37 | 12 | 13 |
| 13 | 14 | 25 | 37 | 11 | 13 |
| 14 | 15 | 25 | 37 | 10 | 13 |

**Table S2.** Melting point and composition of some electrolytes

| **Electrolyte** | **Components (mol%)** | **Cationic elements** | **Experimental m.p. (^◦^C)** |
| --- | --- | --- | --- |
| LiF-LiCl-LiBr | 22–31–47 | Li | 458.8 |
| LiCl-LiBr-NaF | 25–62–13 | Li,Na | 432.5 |
| LiCl-LiBr-KBr | 25–37–38 | Li,K | 323.2 |
| LiF-LiCl-LiBr-KBr-RbBr | 13-25-37-12-13 | Li,Rb | 282.7 |
| LiF-LiCl-LiBr-KBr-CsBr | 13-25-37-12-13 | Li,Cs | 229.5 |

**Table S3.** Thermophysical Properties of Partial Components in Thermal Batteries

| **Components** | **Material** | **Density**  **ρ/(g·cm^-3^)** | **Thermal Conductivity (W·m^-1^·K^-1^)** |
| --- | --- | --- | --- |
| Cathode A | FeS_2_+Electrolyte A | 2.39 | 1.08 |
| Cathode B | FeS_2_+Electrolyte B | 2.55 | 1.19 |
| Electrolyte A | LiCl-LiBr-KBr | 2.02 | 1.17 |
| Electrolyte B | LiF-LiCl-LiBr-KBr-CsBr | 2.28 | 1.28 |
| Anode | LiSi | 0.94 | 5.7 |
| Heat pellet | Fe/KClO_4_ | 3.55 | 22 |
| Current collector | Ni | 8.73 | 71.4 |
